# Standing balance test predicts the Berg Balance Scale score in patients with stroke using principal component analysis

**DOI:** 10.1038/s41598-025-99710-5

**Published:** 2025-05-21

**Authors:** Jieun Cho, Sunghe Ha, Jooyoung Lee, Minsuk Kim, Hogene Kim

**Affiliations:** 1 Translational Research Centre on Rehabilitation Robots, National Rehabilitation Centre, Ministry of Health & Welfare, Seoul, South Korea; 2 Department of Sports Rehabilitation Medicine, Kyungil University, Gyeongsangbuk-do, Gyeongsan-si, South Korea; 3 Department of Applied Statistics, Chung-Ang University, Seoul, South Korea; 4 Department of Mechanical Engineering, University of Michigan, Ann Arbor, MI USA

**Keywords:** Stroke, Balance impairment, Berg Balance Scale, Principal component analysis, Kinematics, Stroke, Experimental models of disease, Translational research

## Abstract

A comprehensive analysis integrating kinematic, kinetic, and electromyographic data to evaluate balance impairments in patients with stroke is lacking. We investigated balance disparities in patients with balance impairment following stroke using principal component analysis (PCA). The complete waveforms of lower-limb-joint angles, centre of pressure, and muscle activity in 43 stroke patients during four Berg Balance Scale (BBS) standing balance tasks were analysed. Multiple regression analysis using principal components (PCs) was conducted to predict BBS scores. Thirteen patients had balance impairments (BBS score < 45). Significant differences in bilateral standing PCs were observed between patients with and without balance impairments during the standing balance tasks (*p* < 0.2). The strongest predictor of BBS score was the performance of the paretic leg during quiet standing with open eyes (*p* < 0.01). Key contributors to balance impairment included bilateral sagittal plane ankle and pelvic joint angles, bilateral vertical ground response forces, and paretic plantar-flexor activation across all standing tasks. These findings highlight that postural control of the paretic limb is a key determinant of balance ability, with distinct balance strategies observed across ability levels. Additionally, PCA effectively quantified balance impairments, revealing significant associations with Fugl-Meyer lower extremity, ankle joint range of motion, and strength. These results emphasize the role of sagittal plane postural control and plantar-flexor activation in stability and suggest that PCA may be a valuable tool for developing targeted rehabilitation strategies.

## Introduction

Stroke survivors with both motor and sensory deficits often experience profound challenges in movement, significantly impacting their quality of life^1^. One of the most critical consequences of these impairments is an increased risk of falls, which further restricts independence and daily function. Studies indicate that 56.2% of stroke survivors are at high risk for falls, with a fall rate of 8.9 per 1000 patients per day^2^. Among the key contributors to these falls is impaired balance control, often characterized by weight-bearing asymmetry—a tendency to over-rely on the nonparetic limb, which leads to increased postural sway and reduced postural stability^3,4^. Prior research suggests that the paretic limb plays a diminished role in postural stabilization^5^ and that stroke patients with postural instability develop compensatory reliance on the nonparetic limb^6,7^. Additionally, individuals with bilateral standing instability exhibit reduced kinetic energy during weight transfer tasks, such as sit-to-stand (STS) transitions, further contributing to balance impairments^8,9^. Given these challenges, restoring bilateral balance control remains a primary goal in stroke rehabilitation.

The Berg Balance Scale (BBS) is widely recognized as the gold standard for assessing balance impairments in stroke rehabilitation^10^. It is commonly used to evaluate postural control recovery and predict fall risk^11^. However, its reliability as a predictive tool remains debated due to inconsistent findings in the literature. While some studies report significant correlations between BBS scores and functional mobility outcomes or fall risk^12,13^, others indicate that BBS fails to predict single or recurrent falls, particularly in community-dwelling stroke survivors^14^, and has limited predictive power in acute-care settings^15^. Beyond its predictive limitations, BBS may not always capture subtle but clinically relevant balance improvements, especially those associated with community reintegration and participation in daily activities^16,17^. Balance recovery after stroke is not solely determined by paretic limb function, but rather by complex interactions between both limbs and other compensatory mechanisms^18^. Studies suggest that rehabilitation programs incorporating both paretic and nonparetic limb involvement are more effective in improving balance and functional outcomes^19^, highlighting the need for a more comprehensive understanding of balance control. Although BBS includes a diverse set of items designed to assess both static and dynamic balance, its clinical utility is further challenged by its floor and ceiling effects, which limit its sensitivity to small functional gains in patients with severe impairments^20,21^ or mild impairments^16^. Moreover, it remains unclear how individual BBS components contribute to overall balance ability or which biomechanical factors play a crucial role in specific balance tasks. Identifying these key biomechanical determinants would not only enhance the clinical interpretation of BBS scores but also provide valuable insights for optimizing rehabilitation strategies aimed at improving both static and dynamic balance recovery in stroke patients.

Recent biomechanical studies have increasingly utilized principal component analysis (PCA) to examine movement characteristics. PCA allows for the deconstruction of complex full-body motion trajectories into distinct motion components, thereby facilitating the identification of key movement patterns across different groups and conditions^22–25^. In stroke research, PCA has been used to extract independent gait characteristics from spatiotemporal, kinematic, and kinetic metrics^22,23^. Several studies have demonstrated that joint kinematic variability is greater in fallers than in non-fallers^24^ and that movement complexity increases with standing balance task difficulty^25^. More recently, joint variability in both paretic and nonparetic limbs was shown to vary depending on the severity of balance impairments in stroke patients^26^. Despite these advancements, most PCA studies have primarily focused on gait analysis, with limited application to standing balance control. Moreover, no studies have systematically integrated multiple biomechanical variables to assess standing balance impairment in stroke patients.

Therefore, this study aims to identify key biomechanical determinants of balance impairments in stroke patients through PCA and evaluate their association with clinical balance measures. By applying PCA to standing balance tasks, this study seeks to quantify postural control characteristics that distinguish individuals with varying balance abilities. This analytical approach offers an objective means of assessing balance deficits, which may contribute to the identification of relevant biomechanical factors for targeted rehabilitation interventions. Accordingly, this study tests the following hypotheses: (1) PCA-derived balance characteristics predict BBS scores in stroke patients; (2) PCA-derived principal components (PCs) effectively differentiate between high and low balance ability groups; and (3) PCA-derived components exhibit significant correlations with clinical variables.

## Results

### Participant selection

**Fig. 1:**
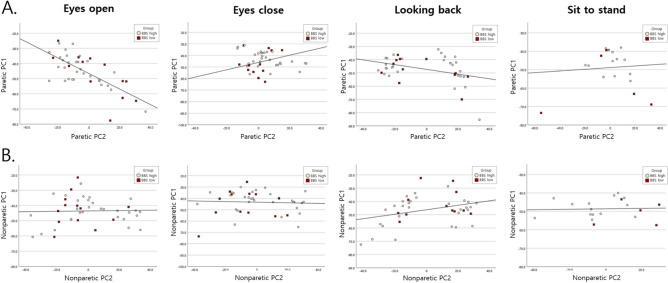
Flow diagram.

Of the 46 participants recruited for inpatient rehabilitation, 43 were included in this study. Three participants were excluded due to insufficient functional ability independently perform the STS movement or because of data loss during movement analysis (Fig. 1).

### Baseline comparison

Among the 43 participants included in the study, 14 (30.2%) exhibited balance impairments and were classified into the BBS-low group. The baseline characteristics of the BBS-high and BBS-low groups are summarized in Table 1. The demographic and clinical characteristics of the BBS-low group were as follows: age (57.8 ± 9.0 years), sex distribution (male:female = 11:2), paretic side (right:left = 8:5), height (169.3 ± 7.9 cm), weight (70.5 ± 8.1 kg), Body Mass Index (24.6 ± 2.3 kg/m^2^), time since stroke onset (10.2 ± 11.7 months), and spasticity score (0:1:1 + :2 = 0:3:10:0). No significant differences in these baseline characteristics were observed between the BBS-high and BBS-low groups. The BBS-low group exhibited significantly lower ankle dorsiflexion range of motion (ROM), isometric contraction force of the ankle dorsiflexor and ankle invertor, BBS scores, and Mini-Mental State Examination scores compared to the BBS-high group (p < 0.05). Additionally, the Fall Efficacy Scale scores were significantly higher in the BBS-low group than in the BBS-high group (p < 0.05), indicating greater fear of falling.

**Table 1:** Demographics of participants.

Variables		Total	BBS-high (n = 30)	BBS-low (n = 13)	*p*-values
Age (years)		52.6 ± 12.4	50.3 ± 13.2	57.8 ± 9.0	0.017
Sex (M:F)		34:9	23:7	11:2	0.567
Paretic side (R:L)		21:22	13:17	8:5	0.284
Height (cm)		170.0 ± 7.3	170.3 ± 7.1	169.3 ± 7.9	0.709
Weight (cm)		69.4 ± 8.3	69.0 ± 8.4	70.5 ± 8.1	0.597
BMI ($\text{kg}/{\text{m}}^{2}$)		24.0 ± 2.2	23.8 ± 2.2	24.6 ± 2.3	0.280
Time since stroke (month)		9.5 ± 8.8	9.2 ± 7.5	10.2 ± 11.7	0.722
Modified Ashworth scale (0:1:1 + :2)		2:11:29:1	2:8:19:1	0:3:10:0	0.427
Range of motion (degree)	Dorsiflexion	14.3 ± 7.4	15.9 ± 7.3	10.7 ± 6.5	0.033*
	Plantar-flexion	132.7 ± 9.3	133.3 ± 9.8	131.2 ± 8.0	0.508
	Inversion	23.5 ± 4.4	24.0 ± 4.5	22.4 ± 3.9	0.284
	Eversion	21.0 ± 3.8	21.5 ± 3.5	19.8 ± 4.3	0.167
Isometric contraction force (N)	Dorsiflexor	12.6 ± 4.6	13.5 ± 4.8	9.6 ± 4.4	0.014*
	Plantar-flexor	14.7 ± 4.8	15.3 ± 5.1	12.3 ± 5.1	0.083
	Inverter	8.5 ± 2.6	9.0 ± 2.3	6.8 ± 3.7	0.020*
	Evertor	8.1 ± 4.2	8.7 ± 4.6	6.2 ± 3.1	0.079
Fugl-Meyer (score)		18.0 ± 4.1	18.4 ± 4.7	16.8 ± 2.1	0.249
Berg Balance Scale (score)		47.4 ± 5.4	50.2 ± 2.5	40.8 ± 4.5	< 0.001*
Standing still with eyes open		4.0 ± 0.3	4.0 ± 0.0	4.0 ± 0.0	1.000
Standing still with eyes closed		4.0 ± 0.2	4.0 ± 0.0	3.9 ± 0.3	0.129
Looking back		3.3 ± 0.5	3.4 ± 0.5	3.0 ± 0.4	0.020*
Sit-to-stand		3.8 ± 0.5	4.0 ± 0.0	3.6 ± 0.8	0.007*
Timed up and go (sec)		29.2 ± 15.4	24.3 ± 12.5	38.2 ± 18.9	0.007*
Fall efficacy scale (score)		49.0 ± 28.3	43.1 ± 29.2	62.6 ± 21.1	0.036*
Mini-mental state estimation (score)		27.7 ± 2.9	28.3 ± 2.7	26.3 ± 3.0	0.034*
Walking speed ($m/sec$)		0.4 ± 0.2	0.5 ± 0.2	0.2 ± 0.1	< 0.001*

The “*” symbol indicates significant differences between BBS-high and BBS-low groups (*p < 0.05). BMI *Body Mass Index*, *BBS* Berg balance scale.

### Multiple regression analysis using PCs and their relative contributions to BBS scores

The results of the multiple linear regression analysis are shown in Table 2. This analysis identified the main determinants of BBS score to be paretic PC3 and PC2 in the eyes open (EO) condition (β = − 0.637, *p* < 0.001 and β = -0.485, *p* = 0.004, respectively). The adjusted coefficient of determination (R^2^) was 0.635, indicating a strong explanatory power of the model. Variance inflation factor ranged from 1.000 to 1.003, indicating the absence of multicollinearity. The regression model was statistically significant (*p* < 0.001).

**Table 2:** Multiple regression analysis to predict Berg balance score.

		B	SE	β	T	*P*
Model 1	(Constant)	47.476	0.833		56.978	< 0.001
	Paretic PC3 in eyes open	− 0.186	0.051	− 0.664	− 3.664	0.002
Model 2	(Constant)	46.661	0.697		66.985	< 0.001
	Paretic PC3 in eyes open	− 0.178	0.040	− 0.637	− 4.472	< 0.001
	Paretic PC2 in eyes open	− 0.174	0.051	− 0.485	− 3.404	0.004

Model 1: BBS = 47.476–0.186*Paretic PC3 in eyes open (${R}^{2}$ = 0.441, Adju-${R}^{2}$ = 0.408, F = 13.428, *P* = 0.002); Model 2: BBS = 46.661–0.178*Paretic PC3 in eyes open-0.174*Paretic PC2 in eyes open (${R}^{2}$ = 0.676, Adju-${R}^{2}$ = 0.635, F = 11.585, *P* = 0.004). *PC* principal component, *BBS* Berg balance score, *SE* standard error.

### Comparison of PCs between the BBS-high and -low groups

PCA identified 42 PCs in the EO condition, 43 in eyes closed (EC), 40 in looking back (LB), and 18 in STS, accounting for over 99% of the movement characteristics. Table 3 presents the explained variance, means, standard deviations (SDs), and effect sizes (Cohen’s d) for the first three PCs (PC1–PC3), which demonstrated the highest explanatory power. Additional PCs, each explaining > 1% of the total variance are presented in Supplementary Tables S1a–S1d. After false discovery rate (FDR) correction, no statistically significant differences were observed between the BBS-high and BBS-low groups. However, effect size analysis revealed 14 PCs with moderate to large effect sizes (Cohen’s d ≥ 0.5), suggesting notable differences in movement characteristics between groups despite the lack of statistical significance.

**Table 3:** Results of principal component analysis.

Categories			Explained Variance (%)	Cumulative (%)	BBS-high (mean ± SD)	BBS-low (mean ± SD)	*q*-values	Cohan’s *d*
Eyes open	P	PC1	59.3	59.3	− 44.2 ± 10.1	− 47.3 ± 18.8	0.678	0.205
		PC2	7.4	66.7	− 5.9 ± 14.7	4.5 ± 21.4	0.252	**0.567**†
		PC3	6.2	72.9	− 2.7 ± 11.8	4.4 ± 28.5	0.334	0.326
	NP	PC1	43.6	43.6	− 43.8 ± 8.8	− 43.1 ± 11.5	0.972	0.068
		PC2	8.0	51.6	2.7 ± 12.8	− 5.9 ± 11.6	0.285	**0.704**†
		PC3	5.5	57.1	− 1.8 ± 19.7	− 1.0 ± 19.9	0.893	0.040
		P15	1.1	84	− 1.8 ± 6.2	4.2 ± 12.0	0.084	**0.628†**
Eyes close	P	PC1	48.4	48.4	− 43.3 ± 8.5	− 50.6 ± 23.9	0.154	0.407
		PC2	5.8	54.2	5.1 ± 9.6	− 5.8 ± 40.0	0.151	0.375
		PC3	5.0	59.2	− 5.2 ± 15.4	5.3 ± 24.0	0.113	**0.520**†
		PC6	2.8	69.3	2.5 ± 10.8	− 6.9 ± 17.0	0.162	**0.660**†
	NP	PC1	43.6	43.6	− 42.5 ± 7.3	− 45.4 ± 16.2	0.403	0.231
		PC2	7.8	51.4	3.1 ± 14.9	− 7.8 ± 12.0	0.148	**0.806**†
		PC3	5.2	56.5	− 1.0 ± 18.6	0.2 ± 18.9	0.639	0.064
		PC16	1.1	84.8	− 1.2 ± 1.0	2.6 ± 2.8	0.144	**1.807†**
Look Back	P	PC1	50.9	50.9	− 50.7 ± 13.7	− 45.3 ± 3.5	0.697	**0.540**†
		PC2	9.5	60.5	− 5.8 ± 21.8	− 1.0 ± 22.8	0.966	0.215
		PC3	4.0	64.4	2.7 ± 15.3	− 5.3 ± 12.0	0.618	**0.582**†
		PC4	3.4	67.9	7.4 ± 16.3	− 7.2 ± 4.6	0.056	**1.219†**
	NP	PC1	49.3	49.3	− 43.7 ± 10.7	− 43.4 ± 8.8	0.944	0.031
		PC2	9.8	59.1	8.9 ± 18.9	5.5 ± 22.8	0.905	0.162
		PC3	6.4	65.5	− 12.1 ± 11.9	− 7.8 ± 11.1	0.859	0.374
Sit-to-stand	P	PC1	56.7	56.7	− 45.8 ± 4.9	− 57.5 ± 6.3	0.399	**2.073**†
		PC2	7.2	63.9	1.3 ± 15.8	− 2.6 ± 15.1	0.810	0.252
		PC3	5.2	69.0	− 6.4 ± 14.0	1.0 ± 22.3	0.401	0.397
	NP	PC1	54.4	54.4	− 47.9 ± 6.3	− 50.8 ± 15.6	0.538	0.244
		PC2	7.0	61.4	− 5.6 ± 33.5	16.0 ± 10.7	0.203	**0.869**†
		PC3	5.9	67.2	− 2.7 ± 25.7	7.3 ± 6.9	0.453	**0.531**†
		PC15	1.3	95.6	− 2.1 ± 7.8	4.5 ± 5.6	0.133	**0.972**†

q-values represent *p*-values adjusted using the false discovery rate (FDR) correction. The symbol '†' indicates a moderate to high effect size according to Cohen’s *d*.

*P* paretic, *NP* nonparetic, *PC* principal component, *BBS* Berg balance scale, *SD* standard deviation.

### Linear relationship between PCs and clinical variables

Spearman’s correlation coefficients between clinical assessments and PCs are presented in Table 4, with only significant correlations after FDR correction reported. In the paretic limb, PCs in the EO condition were negatively correlated with sex (r = − 0.461), paretic side (r = − 0.371), Fugl-Meyer lower extremity (FM-L, r = − 0.421), and BBS (r = -0.388). In the LB condition, paretic PCs were positively correlated with the paretic side (r = 0.866). For the nonparetic limb in LB, PCs were negatively correlated with ROM of eversion (r = − 0.476), sensation (r = − 0.390), and Fall Efficacy Scale (r = − 0.487), while being positively correlated with Timed Up and Go (TUG, r = 0.421). In the STS condition, paretic PCs were negatively correlated with time since stroke (r = − 0.566), ROM of eversion (r = − 0.676), and ROM of inversion (r = − 0.507), while being positively correlated with weight (r = 0.539), ROM of dorsiflexion (r = 0.642), muscle strength of dorsiflexion (r = 0.517), muscle strength of inversion (r = 0.725), and FM-L (r = 0.538). Nonparetic PCs in STS were negatively correlated with ROM of inversion (r = − 0.663) and positively correlated with muscle strength of inversion (r = 0.492).

**Table 4:** Correlations between principal components of standing balance tasks and clinical measurements.

Conditions		Eyes open		Eyes close		Looking back		Sit-to-stand	
Side		P	NP	P	NP	P	NP	P	NP
General	Sex	− .461	–	–	–	–	–	–	–
	Paretic side	− .371	–	–	–	.866	–	–	–
	Height	–	–	–	–	–	–	–	–
	Weight	–	–	–	–	–	–	.539	–
	Time since stroke	–	–	–	–	–	–	− .566	–
Paretic Ankle	ROM	–	–	–	–	–	EV (− .476)	DF (.642) EV (− .676) IV (− .507)	IV (− .663)
	Strength	–	–	–	–	–	–	DF (.517) IV (.725)	IV (.492)
Clinical Measures	MAS	–	–	–	–	–	–	–	–
	Sensation	–	–	–	–	–	− .390	–	–
	FM-L	− .421	–	–	–	–	–	.538	–
	BBS	− .388	–	–	–	–	–	–	–
	TUG	–	–	–	–	–	.421	–	–
	Fall Efficacy Scale	–	–	–	–	–	− .487	–	–

Only significant differences after false discovery rate (FDR) correction are indicated based on Spearman correlation analysis. *P* paretic, *NP* nonparetic, *ROM* range of motion, *DF* dorsiflexion, *EV* eversion, *IV* inversion, *MAS* modified Ashworth scale, *PF* plantar-flexion, *FM-L* Fugl-Meyer lower extremity, *BBS* Berg balance scale, *TUG* timed up and go, *FES* fall efficacy scale.

### Characteristics of PC1 and PC2 of the paretic and nonparetic sides

**Fig. 2:**
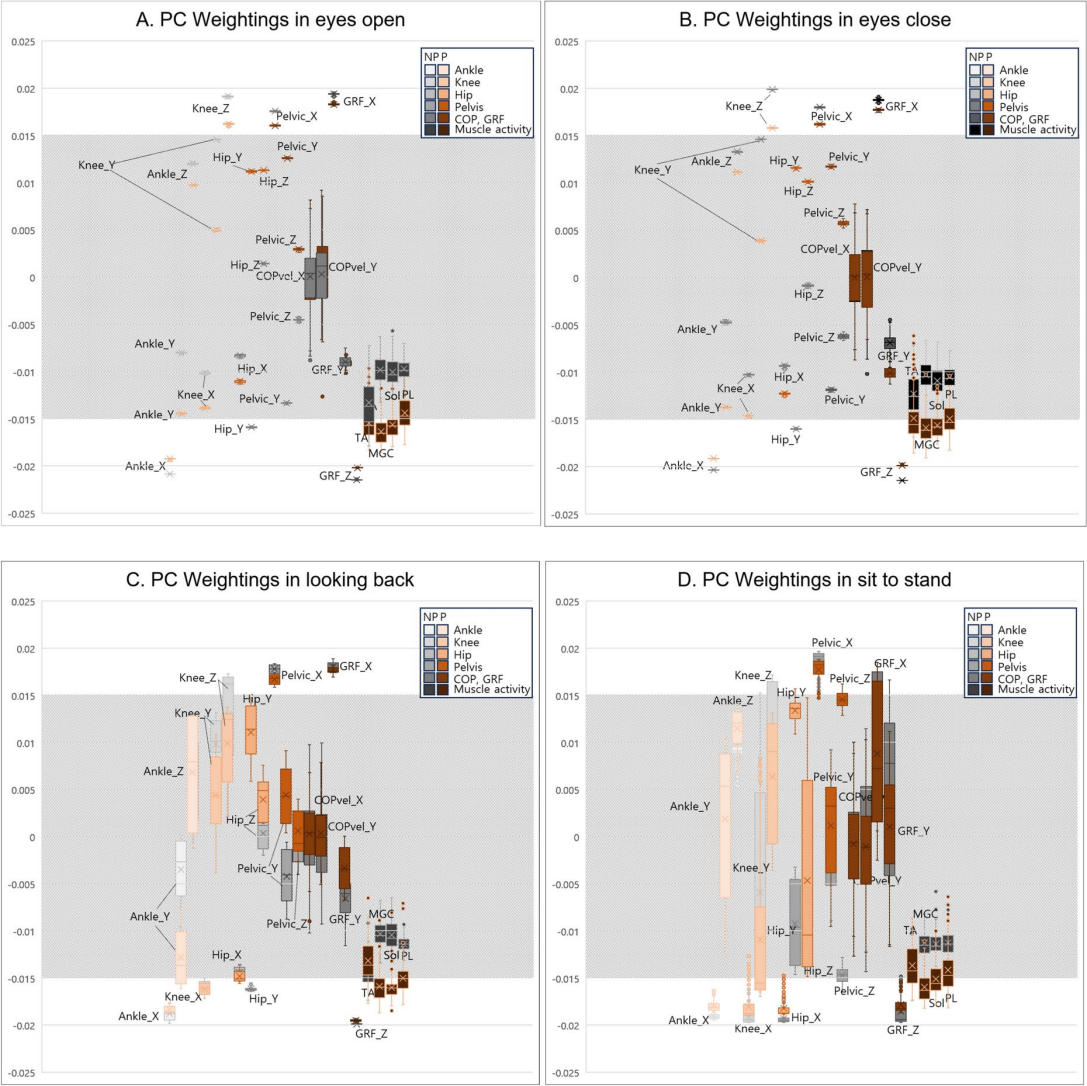
Biplots of PCs for patients with stroke, illustrating the balance impaired group (BBS-low) and balance non-impaired group (BBS-high) for the (**A**) paretic side and (**B**) nonparetic side during each balance task. PC, principal component; BBS, Berg Balance Scale.

Variables with high PC scores were plotted on two simple PC1 and PC2 scatter plots (Fig. 2). For both EO and EC conditions, the scatter plot distribution and the trend line slope of the paretic side was distinguishable from that of the nonparetic side. However, for both LB and STS tasks, the scatter plots of the paretic and nonparetic groups were more similar, with minimal distinction in their distribution or trend of line slopes.

### Contributions of PCs in standing balance conditions

**Fig. 3:**
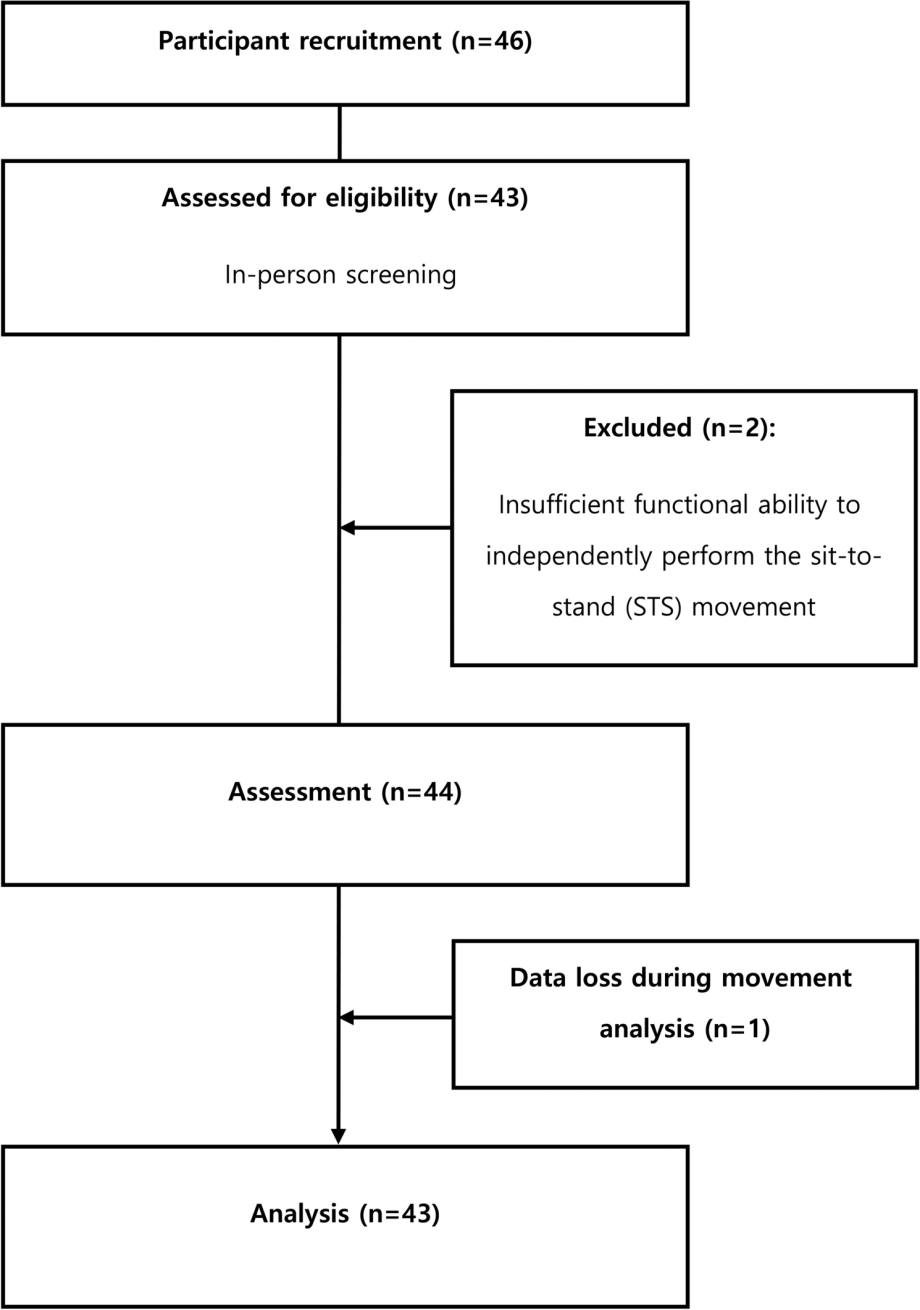
Box-and-whisker plots showing the variation of PC1 loading values (weightings) for patients with stroke. The study posited that variables with absolute values greater than 0.015, indicated by the range of white backgrounds, significantly influenced the performance of each task, exhibiting high explanatory power: (**A**) open eyes, (**B**) close eyes, (**C**) look back, and (**D**) sit-to-stand. PC, principal component; P, paretic; NP, nonparetic; COP, centre of pressure; GRF, ground reaction force; COPvel, velocity of centre of pressure; TA, tibialis anterior; MGC, medial gastrocnemius; Sol, soleus; PL, peroneus longus.

The box-and-whisker plot shows the magnitude of weightings for PC1 as having the highest explanatory power (Fig. 3). The box-and-whisker plots revealed that paretic and nonparetic PC1 had similar loading values in all standing balance tasks. Additionally, EO and EC had similar weighting distributions, while LB and STS had a wider weighting distribution than standing still (EO and EC). Variables with the highest contributions in EO and EC, both positive and negative, above 0.015 (Corresponds to the white background in the illustration) were ankle angle_x, knee angle_z, pelvic angle_x, ground reaction forces (GRF)_x, and GRF_z in both paretic and nonparetic PC1. For paretic PC1, muscle activation of medial gastrocnemius (MGC) and soleus was also included, and for nonparetic PC1, hip angle_y was included in EO and EC. The variables that made the highest contributions in LB (weightings >  ± 0.015, white background in the illustration) were ankle angle_x, knee angle_x, pelvic angle_x, GRF_x, and GRF_z. Additionally, for paretic PC1, muscle activation of the MGC and soleus were included in LB. In STS, the variables with the highest contributions (weightings >  ± 0.015, white background in the illustration) were ankle angle_x, knee angle_x, hip angle_x, pelvic angle_x, and GRF_z. Furthermore, for paretic PC1, muscle activation of the MGC was included in STS.

## Discussion

This study utilized PCA to assess balance impairments in stroke patients and their association with clinical balance measures. Multiple regression analysis identified paretic PC3 and PC2 in the EO condition as significant predictors of BBS scores (adjusted R^2^ = 0.635, *p* < 0.001). While no statistically significant group differences were found after FDR correction, effect size analysis revealed 14 PCs with moderate to large effects, indicating distinct postural control patterns. Correlation analysis demonstrated significant associations between specific PCs and clinical measures, including FM-L, joint ROM, and functional mobility. During all standing balance tasks, the bilateral sagittal plane ankle and pelvic joint angles, bilateral vertical GRF, and muscle activation of the paretic plantar-flexor were found to be significant contributors. These findings suggest that PCA-derived balance characteristics provide objective insights into postural control deficits and may aid in developing targeted rehabilitation strategies for stroke patients, offering insights into the following balance characteristics.

First, prediction of BBS scores based on PCA-derived balance characteristics. The results of multiple regression analysis revealed that paretic PC3 and PC2 during the EO condition were significant determinants of BBS scores (β = − 0.637, *p* < 0.001; β = − 0.485, *p* = 0.004, respectively). The model demonstrated strong explanatory power (adjusted R^2^ = 0.635), suggesting that postural control in the paretic limb during quiet standing plays a crucial role in overall balance ability. These findings align with previous research indicating that balance impairments following stroke are strongly influenced by the degree of motor impairment in the paretic limb, as well as the ability to control weight-bearing asymmetry^27^. Postural control strategies in stroke patients often involve compensatory reliance on the nonparetic limb due to impaired weight-shifting ability on the paretic side. This compensatory mechanism has been reported in previous studies, where individuals with severe balance impairments exhibited greater weight-bearing asymmetry, reduced lateral stability, and increased fall risk^28,29^. The inability to effectively recruit paretic limb postural muscles may lead to greater reliance on proximal and nonparetic limb support, further reinforcing asymmetrical balance patterns^30^. Importantly, the present study included participants with lower FM-LE scores (mean 18.0 ± 4.1), indicating reduced mobility function. Given that an FM-LE score of 21 or higher is typically associated with greater mobility in chronic stroke survivors^31^, it is possible that the participants in this study had more severe motor impairments, which may have influenced their balance control strategies. Previous studies suggest that stroke survivors with milder motor impairment may rely less on the nonparetic limb and demonstrate better postural control on the paretic side^32^. Thus, caution is required when applying these findings to individuals with milder impairment, as their balance mechanisms may differ. Future studies should examine whether similar relationships are observed in patients with higher FM-LE scores and greater motor function.

Second, Differentiation of Balance Ability Groups Based on PCA-Derived PCs. This study suggests that PCA-derived PCs can distinguish between stroke patients with high and low balance ability. Although no statistically significant differences were observed between the BBS-high and BBS-low groups, effect size analysis revealed 14 PCs with moderate to large effect sizes (Cohen’s d ≥ 0.5), indicating meaningful differences in postural control strategies. When assessing clinically meaningful differences, effect sizes should be prioritized over statistical significance, as they provide a clearer measure of the magnitude of observed changes and their practical implications, particularly in rehabilitation research where even small improvements can be functionally significant^33,34^. The presence of these effect sizes suggests that stroke patients with lower balance ability may adopt altered postural control mechanisms to compensate for neuromuscular impairments. Previous studies have shown that individuals with severe balance impairments tend to rely more on the nonparetic limb for weight-bearing and postural adjustments, leading to asymmetrical and inefficient balance control^29^. Greater weight-bearing asymmetry has been associated with increased dynamic control asymmetry and reduced mediolateral sway control^32^, as well as increased center-of-pressure (COP) velocity and greater postural sway^35^. These findings align with the results of this study, supporting the notion that differences in balance ability are linked to distinct postural control strategies. Moreover, the results demonstrate that stroke patients exhibit not only paretic limb deficits but also differences in the utilization of the nonparetic limb depending on their balance ability. Consistent with this, prior research has identified functional deficits in the nonparetic limb, including reduced knee ROM, slower movement speed, impaired proprioception, decreased extensor and flexor strength, and diminished balance ability^36^. Additionally, incorporating motor therapy programs that engage both the nonparetic and paretic limbs has been found to improve balance and functional outcomes in stroke patients^19^. These findings further emphasize that postural control adaptations extend beyond the paretic limb, highlighting the need to consider nonparetic limb function in balance assessments and rehabilitation.

Third, this study analyzed the correlations between PCA-derived PCs and various clinical measures, identifying distinct balance characteristics in stroke patients. During the EO condition, paretic PCs were negatively correlated with FM-L and BBS, suggesting that better motor function and balance ability are associated with reduced balance deviations in the paretic limb. Additionally, the correlation with sex and paretic side indicates that individual factors, such as lesion laterality, may influence balance control strategies. In the LB condition, paretic PCs showed a strong positive correlation with the paretic side, while nonparetic PCs were negatively correlated with ankle eversion ROM, sensation, and fall efficacy, and positively correlated with TUG. These findings suggest that greater limitations in ankle ROM and increased fear of falling may lead to compensatory postural adjustments in the nonparetic limb. During the STS condition, paretic PCs were positively correlated with ankle ROM and strength, while negatively correlated with time since stroke and reduced ankle mobility. This indicates that patients with greater ankle mobility and strength demonstrate more stable balance, whereas those with prolonged post-stroke duration and restricted ankle movement exhibit greater balance deviations. Nonparetic PCs were positively correlated with ankle inversion strength, suggesting that patients with weaker inversion strength may rely more on their nonparetic limb for compensation. These findings emphasize the multifactorial nature of balance control in stroke patients, influenced by motor function and biomechanical constraints, highlighting the need for comprehensive lower limb assessments in future research.

Fourth, distinct postural control strategies depending on task conditions. In the LB and STS tasks, sagittal plane movements of the ankle, knee, pelvis, and hip played a key role, emphasizing multi-joint coordination. In contrast, the EO and EC conditions primarily involved ankle dorsiflexion/plantarflexion and hip adduction/abduction, with GRF and activation of the MGC and soleus being essential for balance control. These findings align with previous research, indicating that sagittal plane movements and ankle muscle activation are crucial for maintaining balance. Studies have shown that bilateral ankle and pelvic movements, along with MG activation, are key contributors to balance control, while increased anterior–posterior COP displacement is associated with poorer balance performance^37^. Additionally, weight-bearing asymmetry serves as a compensatory mechanism, particularly in the anterior–posterior direction, to optimize the role of the nonparetic limb^32^. Muscle activation analysis further demonstrated that MGC activation was significantly associated with ankle, knee, and hip movements, and co-contraction of the gastrocnemius and tibialis anterior played a key role in postural stability^38,39^. This suggests that ankle muscle coordination is essential for balance maintenance and is closely linked to multi-joint movement control. Therefore, rehabilitation programs for stroke patients should incorporate sagittal plane stability and ankle muscle activation strategies to improve balance control.

## Strength and limitations

This study has several notable strengths. First, the balance tasks employed in this study were relatively easy to perform, facilitating a comparison between participants with varying levels of functioning. This facilitated the measurement and comparison of balance characteristics across a range of functional levels in patients. Second, this is the first study to report balance characteristics in stroke patients utilising PCA to facilitate a comprehensive, specific analysis of kinematic, kinetic, and electromyographic data obtained during a balance task performed in a standing position. To gain a better understanding of the postural control mechanism in this populations, this study utilised PCA to generate new variables from multiple data sources to measure standing balance in patients with stroke. All 43 participants in this study achieved a score of 4 on the 'standing still with EO’ item of the BBS. However, PCA revealed significant differences in 'standing still with EO’ when balance impairment was taken into consideration. A recent PCA study revealed that anterior–posterior and medial–lateral measures of postural steadiness in the elderly can distinguish fallers from non-fallers^40^. This indicates that even patients who are able to perform the same standing posture may differ in their specific balance strategies and postural control. In this regard, PCA could be a valuable method for measuring the complexity of postural control movements observed during a specific balance tasks^25^.

Despite its strengths, this study has some limitations. First, the relatively small sample size (n = 43), which may constrain the generalizability of the findings. While previous studies have successfully applied PCA in small sample settings and have demonstrated its methodological robustness through resampling techniques such as the jackknife method^24,41^, caution should be exercised in interpreting the results. Future studies with larger and more diverse populations are warranted to confirm the generalizability and reproducibility of our findings. Second, the distribution of the patients across the two groups, categorised by balance impairment, was uneven. To eliminate the potential impact of unequal group distributions on the outcomes, a statistical technique known as a homogeneity test was used to assess the basic characteristics of the groups. However, future studies with a larger sample size are needed to confirm the results of this study. Third, for safety reasons, the study excluded stroke patients who had severely impaired balance. The mean BBS score of the participants was 47.4 (range 33–54), which may influence the generalizability of the results. Therefore, the results of this study may not be representative of all functional levels of stroke patients, but more applicable to those who are able to stand independently and perform STS tasks. Fourth, this study was performed with only a limited set of stance balance tasks. It has been demonstrated earlier that the BBS scores correlate with step length and swing time asymmetries (*r* = − 0.36 to − 0.63), which suggest spatiotemporal gait asymmetry related to high number of falls being more closely associated with balance measures involving dynamic tasks than static tasks^42^. Therefore, future research should investigate the application of PCA to diverse dynamic balance tasks in relation to falls in stroke patients.

## Conclusion

This study utilized PCA to analyze balance impairments in stroke patients and assess their associations with clinical measures. The results indicate that postural control of the paretic limb during standing with EO is a key determinant of overall balance ability (BBS scores), and stroke patients exhibit different balance strategies depending on their balance ability. PCA-derived PCs were significantly correlated with FM-L, ankle ROM, and strength, highlighting the importance of sagittal plane postural control and plantar-flexor activation in balance regulation. These findings suggest that balance impairments in stroke patients are influenced by multiple factors, including motor function and biomechanical constraints, emphasizing the need for comprehensive balance assessments and future research incorporating diverse balance tasks. Future research needs to apply PCA to a broader range of dynamic balance tasks, using larger sample sizes, and including patients with different functional levels.

## Methods

### Design

This study employed a cross-sectional design and was conducted at a single centre.

### Participants

A total of 46 individuals with chronic stroke were recruited from inpatient clinics at the National Rehabilitation Centre, Seoul, South Korea, for voluntary participation in the study. To ensure a systematic and unbiased recruitment process, we employed a multi-step approach consisting of public advertisement, telephone screening, and in-person eligibility verification.

First, study recruitment posters were displayed in the inpatient rehabilitation wards of the National Rehabilitation Centre. The posters provided an overview of the study’s objectives, inclusion criteria, and contact information for interested individuals. Patients who expressed interest in participating contacted the research team via telephone, at which point a preliminary screening interview was conducted. During this initial screening, participants were asked about their medical history, time since stroke onset, ability to maintain an independent standing position, and any orthopaedic conditions or pain that might interfere with participation. If individuals met the preliminary inclusion criteria, they were invited to an in-person screening session at the rehabilitation centre. Only individuals who met all inclusion criteria and none of the exclusion criteria were enrolled in the study. All participants were right-side dominant and exhibited hemiplegic gait patterns that affected their postural balance and ambulation. The final inclusion criteria were as follows: (1) individuals who had experienced a stroke more than 6 months prior, (2) ability to maintain a standing position independently for at least 30 s, (3) absence of orthopaedic problems or significant pain in the lower extremities, and (4) hemiplegia on the right or left side of the body. Patients with poor visual depth perception, inability to control their posture or limbs (Modified Ashworth Scale score > 3), and cognitive deficits that influenced their ability to understand the study and follow instructions (Mini-Mental State Examination score < 20) were excluded. Since participants were self-selected based on voluntary response to the study advertisement, there is a possibility of selection bias, as individuals who were more motivated to participate in rehabilitation-based research may have been overrepresented. Additionally, since all participants were recruited from a single rehabilitation centre, the findings may not fully generalize to individuals undergoing rehabilitation in different clinical settings.

The experimental protocol was approved by the ethical review board at the National Rehabilitation Centre, Seoul, South Korea (IRB number: NRC 2022-02-014, 29/03/2022), and all participants provided written informed consent before participating. The research complied with the principles of the Declaration of Helsinki.

### Definition of balance impairment

BBS comprises a set of 14 items for the assessment of functional activities in daily life tasks and is considered the criterion standard for the assessment of static and dynamic balance abilities. These activities are classified from 0 (unable) to 4 (independent). A cut-off score of < 45 in BBS have been determined to predict the risk of falls, length of stay and discharge destination for inpatient rehabilitation, and degree of improvement to achieve community walking speed in stroke patients^10,43^. BBS demonstrates acceptable sensitivity (91%,) and specificity (82%) for predicting the risk of falls^44^. In this study, therefore, a BBS cut-off score of < 45 was considered as indicative of balance impairment and classified as the BBS-low group, and a BBS score of > 45 was classified as the BBS-high group.

### Clinical assessments

All measurements were performed by skilled physiotherapists. For assessing ankle function, the passive ROM of the paretic ankle was measured using a portable goniometer (Chattanooga, CA, USA). The average values of three measurements were recorded for the maximum passive ROM of dorsiflexion, plantar-flexion, eversion, and inversion. To measure ankle muscle strength, the isometric contraction force of the paretic ankle muscle was measured using a portable manual muscle strength tester. The isometric strength of the ankle dorsiflexor, plantar-flexor, invertor, and evertor was measured for 5 s, and the maximum value was recorded. The motor domain of the FM-L assessment was used to measure motor impairment^45^. This domain includes measurements of movement, coordination, and reflex action for the hip, knee, and ankle. The FM-L is rated on a 3-point ordinal scale (0 = cannot be performed, 1 = partially performed, and 2 = fully performed). The maximum possible score for the motor domain of the FM-L assessment is 34, corresponding to full sensorimotor recovery. The Korean version of the Fall Efficacy Scale was used to ascertain the patient’s level of confidence in performing activities of daily living^46^. This self-report questionnaire contains 10 items, each scored on a scale of 0–10. The total summed score ranges from 0 to 100, with a higher score indicating increased confidence in performing activities of daily living without falling.

### Standing balance measurements

The measurements of standing balance tasks were performed in a quiet room. Three-dimensional (3D) positional data were collected during standing balance task, using reflective markers and a 12-camera 3D motion capture system (VICON, Saint Helens, UK) with a 100 Hz sampling frequency. A total of 23 reflective markers were attached following the guidelines of the Visual 3D software (C-Motion Ubc., Rockville, MD, US). Simultaneously, GRFs and COPs were measured using two force plates (Advanced Medical Technology Inc., Watertown, MA, USA) sampled at 2000 Hz. Additionally, muscle activities were obtained using eight surface electromyography devices (EMG; Delsys Trigno Wireless EMG, Delsys, USA) sampling at 2000 Hz placed on both sides of the TA, MGC, soleus, and peroneus longus. Before the standing balance task trials, the positions of the markers were recorded while the participants were stationary. We used the following four items of BBS that can be assessed in a standing position to better understand the standing balance characteristics^47^: (1) standing still with EO for 30 s; (2) standing still with EC for 30 s; (3) while keeping the lower foot fixed on the force plate, turning to look behind to the left and right (LB); and (4) while keeping the lower foot fixed on the force plate, sit and stand up in the back chair from a standing position (STS). The participants received sufficient training to ensure successful performance of each task. After practice, three successful trials of each of the four standing tasks were recorded.

### Data analyses

Raw motion data were digitally filtered using a zero-lag, fourth-order, low-pass Butterworth filter, with a filter cut-off frequency of 6 Hz. The hip, knee, and ankle joint angles, and the pelvis-link angle during each task cycles were calculated for the x-axis (e.g., flexion–extension), y-axis (e.g., abduction–adduction), and z-axis (e.g., internal–external rotation) using a Cardan sequence of rotations (X–Y-Z) from the trajectories measured in each trial (joint-specific movements for each axis are presented in Supplementary Table S2). EMG data were processed using a 20–400 Hz bandpass filter and rectification and were normalised by maximum voluntary isometric contraction. All joint angles, GRFs, COPs, and muscle activations were time-normalised using the standing balance task duration and divided into 101 variables ranging from 0 to 100%^48^. Therefore, each trial corresponded to a dataset of 116,352 variables (101 time points, four joint angles in three axes, GRFs in three axes, path and velocity of COPs in two axes, muscle activities of four lower limb muscles, including their means, and SDs). Data for the paretic and nonparetic sides were obtained separately. Low-pass filtering, variable calculation (joint and link angles), and time normalisation processes were performed using Visual 3D (https://c-motion.com).

#### PCA

PCA was applied to the correlation matrix of the 116,352 variables calculated from the 215 data points (43 participants in three trials and both legs). PCA was used to simplify the complexity of high-dimensional data (116,352 parameters), while retaining trends and patterns by transforming the data into fewer dimensions, which summarise the key features (variables with high PC scores). The specific PCA procedure was as follows: (1) intra-participant average and SDs were calculated for each time point within the three trials of data obtained from each participant. (2) mean centring was conducted on each of the 116,352 variables using the z-score:

$${Z}_{t}=\frac{({X}_{t}-{\mu }_{t})}{{\sigma }_{t}}$$

where ${Z}_{t}$ refers to the z-score for the parameter *t* (the parameter *t* refers to each of the 101 time-normalised time points), ${X}_{t}$ refers to the raw data of the parameter *t*, ${\mu }_{t}$ refers to the mean of the parameter t for the participant, and ${\sigma }_{t}$ refers to the SD of the parameter t. The value of the parameter *t* ranged between 1 and 116,352. (3) input matrices of 86 data points (43 participants and both lower limbs) by 116,352 variables were constructed. (4) PC vectors (PCVs) were extracted until their cumulative ratio attained approximately 100% for the paretic and nonparetic sides of the total variance. (5) statistical analyses were conducted to identify the main effects of balance impairment on the movement characteristics during standing balance task represented by the PCVs. (6) to compare the paretic and nonparetic conditions, scatter plots were used to demonstrate the variation of PC1 and PC2 during each standing balance task. (7) the box-and-whisker plot was used to illustrate the variation of the PC1 loading values (weightings), which had the largest variation, to show their variation of each component (joint kinematics, COP, and muscle activities) in each standing balance task. The magnitude of PC weightings determined their contribution to the PC.

### Statistical analyses

All statistical analyses were performed using SPSS version 22.0 (IBM, Armonk, NY, USA). The normality of baseline data was assessed using the Shapiro–Wilk test, and statistical significance was set at *p* < 0.05. Group comparisons of general characteristics and clinical variables between the BBS-high and BBS-low groups were conducted using independent t-tests or Chi-square tests. To examine whether PCA-derived components predict BBS scores in stroke patients, stepwise multiple linear regression analysis was performed with BBS score as the dependent variable and the first three PCs (PC1–PC3) as independent variables, which demonstrated the highest explanatory power. To determine whether PCA-derived PCs effectively differentiate between high and low balance ability groups, independent t-tests were conducted to compare PCs between the BBS-high and BBS-low groups, following previous studies analyzing principal component differences^24,49^. To control for false positives due to multiple comparisons, FDR correction using the Benjamini-Hochberg (BH) procedure was applied^50^, with statistical significance set at FDR-corrected *p* < 0.05. Additionally, Cohen’s d effect sizes were calculated to assess the magnitude of differences, with thresholds of 0.2 (small), 0.5 (moderate), and 0.8 (large). Lastly, Spearman’s rank correlation coefficients were computed to evaluate the relationship between PCA-derived components and clinical variables, with BH correction applied to adjust for multiple testing effects.

## Supplementary Information

Supplementary Information.

## Data Availability

Supplementary information is available for he research data used in this study. The datasets generated and/or analysed during this study are available from the corresponding author upon reasonable request.
