# Supplementary material for: Standing balance test predicts the Berg Balance Scale score in patients with stroke using principal component analysis
Source: Sci Rep. 2025 May 21;15:17653. doi: 10.1038/s41598-025-99710-5 (PMC12095567; doi:10.1038/s41598-025-99710-5)
Supplement: Supplementary file 1 — Supplementary Information. [file 41598_2025_99710_MOESM1_ESM.docx]

**Supplementary Information**

**Standing balance test predicts the Berg Balance Scale score in patients with stroke: A principal component analysis study**

Jieun Cho^1^, Sunghe Ha^2^, Jooyoung Lee^3^, Minsuk Kim^3^, Hogene Kim^4*^

**This document includes:**

- Supplementary Tables S1 (a-d) and S2

**Supplementary Table S1-a.** Results of principal component analysis (PCA) in **eyes open**. PCA was applied to a correlation matrix of 116,352 variables (101 time points, four angles in three axes, ground reaction force in three axes, two centre of pressure variables in two axes, four muscle activations, means, and standard deviations [SDs]) calculated from the 86 data sets (43 participants and both lower limbs).

| **Categories** | | **Explained Variance**  **(%)** | **Cumulative (%)** | **BBS High**  **(mean±SD)** | **BBS Low (mean±SD)** | ***p*** | **Cohan's *d*** |
| --- | --- | --- | --- | --- | --- | --- | --- |
| Paretic | PC1 | 59.3 | 59.3 | -44.2±10.1 | -47.3±18.8 | 0.484 | 0.205 |
|  | PC2 | 7.4 | 66.7 | -5.9±14.7 | 4.5±21.4 | **0.072*** | 0.567 |
|  | PC3 | 6.23 | 72.93 | -2.7±11.8 | 4.4±28.5 | **0.191*** | 0.326 |
|  | PC4 | 5.49 | 78.42 | -1.5±11.8 | 1.6±33.2 | 0.614 | 0.124 |
|  | PC5 | 3.44 | 81.86 | -2.8±16.4 | 3.9±12.2 | **0.097*** | 0.464 |
|  | PC6 | 2.6 | 84.46 | 0.2±11.7 | 1.0±14.7 | 0.835 | 0.060 |
|  | PC7 | 1.96 | 86.42 | 0.4±11.9 | -3.5±10.3 | 0.363 | 0.350 |
|  | PC8 | 1.79 | 88.21 | 0.7±11.8 | -2.7±7.2 | 0.292 | 0.348 |
|  | PC9 | 1.34 | 89.55 | -1.0 ±10.1 | 2.5±7.8 | 0.313 | 0.388 |
|  | PC10 | 1.31 | 90.86 | -0.5±10.0 | 0.1±11.1 | 0.886 | 0.057 |
|  | PC11 | 1.0 | 91.8 | 0.7±10.4 | -1.5±8.1 | 0.497 | 0.236 |
| Non-paretic | PC1 | 43.6 | 43.6 | -43.8±8.8 | -43.1±11.5 | 0.833 | 0.068 |
|  | PC2 | 8.0 | 51.6 | 2.7±12.8 | -5.9±11.6 | **0.122*** | 0.704 |
|  | PC3 | 5.5 | 57.1 | -1.8±19.7 | -1.0±19.9 | 0.893 | 0.040 |
|  | PC4 | 3.7 | 60.8 | -0.9±9.2 | 4.5±29.5 | **0.163*** | 0.247 |
|  | PC5 | 3.5 | 64.3 | -2.3±15.9 | 4.2±21.1 | **0.191*** | 0.348 |
|  | PC6 | 3.1 | 67.4 | 0.9±13.7 | -2.3±12.7 | 0.430 | 0.242 |
|  | PC7 | 2.8 | 70.2 | -1.0±9.8 | 1.4±8.3 | 0.526 | 0.264 |
|  | PC8 | 2.6 | 72.8 | 0.1±11.5 | -0.9±8.9 | 0.793 | 0.972 |
|  | PC9 | 2.1 | 74.9 | 0.5±10.9 | -0.8±12.8 | 0.710 | 0.109 |
|  | PC10 | 1.9 | 76.9 | -0.3±8.4 | -1.6±9.7 | 0.701 | 0.143 |
|  | PC11 | 1.8 | 78.6 | 0.5±9.2 | -2.0±8.9 | 0.462 | 0.276 |
|  | PC12 | 1.6 | 80.2 | -0.8±9.5 | -0.6±3.1 | 0.957 | 0.028 |
|  | PC13 | 1.4 | 81.6 | -1.1±7.9 | 3.1±9.6 | **0.111*** | 0.478 |
|  | PC14 | 1.2 | 82.8 | -0.6±6.7 | 1.4±11.6 | 0.411 | 0.211 |
|  | PC15 | 1.1 | 84.0 | -1.8±6.2 | 4.2±12.0 | **0.012*** | 0.628 |
|  | PC16 | 1.1 | 85.0 | -0.5±6.0 | 0.8±9.1 | 0.608 | 0.169 |
|  | PC17 | 1.0 | 86.0 | -0.0±6.1 | -0.5±5.0 | 0.760 | 0.090 |

The “*” symbol indicates significant differences between BBS-high and BBS-low groups (**p* < 0.2). *PC* principal component, *BBS* Berg balance scale.

**Supplementary** **Table S1-b.** Results of main principal component analysis in **eyes close**.

| **Categories** | | **Explained Variance**  **(%)** | **Cumulative (%)** | **BBS High**  **(mean±SD)** | **BBS Low (mean±SD)** | ***p*** | **Cohan's *d*** |
| --- | --- | --- | --- | --- | --- | --- | --- |
| Paretic | PC1 | 48.4 | 48.4 | -43.3±8.5 | -50.6±23.9 | **0.154** | 0.407 |
|  | PC2 | 5.8 | 54.2 | 5.1±9.6 | -5.8±40.0 | **0.126** | 0.375 |
|  | PC3 | 5.0 | 59.2 | -5.2±15.4 | 5.3±24.0 | **0.075** | 0.520 |
|  | PC4 | 4.0 | 63.2 | -2.0±14.9 | 1.3±8.5 | 0.505 | 0.272 |
|  | PC5 | 3.3 | 66.5 | 1.4±13.0 | -2.4±9.9 | 0.300 | 0.329 |
|  | PC6 | 2.8 | 69.3 | 2.5±10.8 | -6.9±17.0 | **0.027** | 0.660 |
|  | PC7 | 2.5 | 71.8 | -0.5±12.2 | 3.2±5.4 | 0.364 | 0.392 |
|  | PC8 | 2.2 | 74.0 | 0.7±10.2 | -1.0±8.7 | 0.651 | 0.179 |
|  | PC9 | 1.8 | 75.9 | -0.8±11.7 | 0.4±8.1 | 0.782 | 0.119 |
|  | PC10 | 1.7 | 77.6 | 0.5±14.0 | -0.6±4.0 | 0.782 | 0.107 |
|  | PC11 | 1.5 | 79.1 | 1.1±9.6 | -2.1±2.6 | 0.307 | 0.455 |
|  | PC12 | 1.3 | 80.3 | -0.7±7.4 | 1.1±5.2 | 0.541 | 0.281 |
|  | PC13 | 1.2 | 81.5 | -1.3±8.2 | 2.5±10.3 | **0.092** | 0.408 |
|  | PC14 | 1.2 | 82.7 | -0.4±8.4 | 1.8±5.9 | 0.380 | 0.303 |
|  | PC15 | 1.2 | 83.9 | 0.6±6.9 | -0.6±7.9 | 0.643 | 0.162 |
|  | PC16 | 1.1 | 85.0 | 0.7±9.2 | -2.1±6.2 | **0.182** | 0.357 |
|  | PC17 | 1.0 | 85.9 | -0.9±8.0 | 2.4±3.2 | **0.155** | 0.542 |
| Non-paretic | PC1 | 43.6 | 43.6 | -42.5±7.3 | -45.4±16.2 | 0.470 | 0.231 |
|  | PC2 | 7.8 | 51.4 | 3.1±14.9 | -7.8±12.0 | **0.074** | 0.806 |
|  | PC3 | 5.2 | 56.5 | -1.0±18.6 | 0.2±18.9 | 0.852 | 0.064 |
|  | PC4 | 3.8 | 60.3 | 4.1±12.8 | -4.3±21.9 | **0.122** | 0.468 |
|  | PC5 | 3.3 | 63.6 | 0.5±12.6 | -2.2±15.5 | 0.547 | 0.191 |
|  | PC6 | 3.2 | 66.7 | 0.9±14.6 | -3.3±7.8 | 0.324 | 0.359 |
|  | PC7 | 2.9 | 69.7 | -0.7±12.8 | 0.5±12.4 | 0.788 | 0.095 |
|  | PC8 | 2.5 | 72.2 | -1.3±11.4 | 0.6±12.2 | 0.597 | 0.161 |
|  | PC9 | 2.3 | 74.5 | -0.8±8.5 | 3.4±23.3 | **0.141** | 0.239 |
|  | PC10 | 2.0 | 76.5 | -1.2±9.6 | 3.9±9.7 | **0.148** | 0.528 |
|  | PC11 | 1.7 | 78.3 | -0.4±7.9 | 3.0±7.7 | 0.215 | 0.436 |
|  | PC12 | 1.5 | 79.8 | -0.9±6.7 | 1.7±6.9 | 0.373 | 0.382 |
|  | PC13 | 1.4 | 81.2 | 0.7±6.1 | -0.2±6.7 | 0.717 | 0.140 |
|  | PC14 | 1.3 | 82.5 | 0.1±10.1 | 0.1±4.8 | 0.989 | 0 |
|  | PC15 | 1.3 | 83.8 | 0.2±9.1 | -0.2±7.4 | 0.853 | 0.048 |
|  | PC16 | 1.1 | 84.8 | -1.2±1.0 | 2.6±2.8 | **0.048** | 1.807 |
|  | PC17 | 1.0 | 85.9 | 0.3±9.6 | -1.1±3.1 | 0.634 | 0.196 |
|  | PC18 | 1.0 | 86.8 | 0.1±4.6 | -0.4±3.4 | 0.796 | 0.124 |

The “*” symbol indicates significant differences between BBS-high and BBS-low groups (**p* < 0.2). *PC* principal component, *BBS* Berg balance scale.

**Supplementary** **Table S1-c.** Results of main principal component analysis in **looking back**.

| **Categories** | | **Explained Variance**  **(%)** | **Cumulative (%)** | **BBS High**  **(mean±SD)** | **BBS Low (mean±SD)** | ***p*** | **Cohan’s *d*** |
| --- | --- | --- | --- | --- | --- | --- | --- |
| Paretic | PC1 | 50.9 | 50.9 | -50.7±13.7 | -45.3±3.5 | **0.199** | 0.540 |
|  | PC2 | 9.5 | 60.5 | -5.8±21.8 | -1.0±22.8 | 0.690 | 0.215 |
|  | PC3 | 4.0 | 64.4 | 2.7±15.3 | -5.3±12.0 | 0.265 | 0.582 |
|  | PC4 | 3.4 | 67.9 | 7.4±16.3 | -7.2±4.6 | **0.008** | 1.219 |
|  | PC5 | 2.8 | 70.7 | -1.3±14.3 | -6.3±4.6 | 0.269 | 0.471 |
|  | PC6 | 2.4 | 73.1 | 1.6±14.6 | 0.3±4.3 | 0.770 | 0.121 |
|  | PC7 | 2.3 | 75.4 | 5.2±10.4 | -6.8±11.6 | **0.086** | 1.090 |
|  | PC8 | 2.0 | 77.4 | -3.7±10.4 | -3.9±11.4 | 0.983 | 0.018 |
|  | PC9 | 1.7 | 79.1 | 0.9±6.1 | -0.8±7.8 | 0.674 | 0.243 |
|  | PC10 | 1.5 | 80.6 | -0.2±10.1 | -1.8±8.0 | 0.740 | 0.176 |
|  | PC11 | 1.4 | 82.0 | 1.0±9.0 | -2.4±9.0 | 0.497 | 0.378 |
|  | PC12 | 1.2 | 83.2 | -2.3±6.4 | -6.2±4.5 | **0.172** | 0.705 |
|  | PC13 | 1.2 | 84.4 | 1.4±9.2 | 3.3±0.7 | 0.454 | 0.291 |
|  | PC14 | 1.1 | 85.5 | 0.8±7.8 | 1.7±7.2 | 0.816 | 0.120 |
|  | PC15 | 1.1 | 86.5 | 1.0±7.7 | 1.8±5.7 | 0.812 | 0.118 |
| Non-paretic | PC1 | 49.3 | 49.3 | -43.7±10.7 | -43.4±8.8 | 0.944 | 0.031 |
|  | PC2 | 9.8 | 59.1 | 8.9±18.9 | 5.5±22.8 | 0.776 | 0.162 |
|  | PC3 | 6.4 | 65.5 | -12.1±11.9 | -7.8±11.1 | 0.491 | 0.374 |
|  | PC4 | 3.1 | 68.6 | 1.6±9.2 | -10.4±13.4 | **0.118** | 1.044 |
|  | PC5 | 2.6 | 71.2 | 1.5±10.2 | 5.7±8.4 | 0.389 | 0.450 |
|  | PC6 | 2.1 | 73.3 | -2.0±10.2 | 1.7±7.2 | 0.402 | 0.419 |
|  | PC7 | 2.0 | 75.3 | -4.4±7.1 | -0.0±8.8 | 0.356 | 0.550 |
|  | PC8 | 1.9 | 77.2 | -1.9±8.0 | 7.1±10.3 | **0.129** | 0.976 |
|  | PC9 | 1.5 | 78.8 | 1.9±7.4 | -3.1±17.4 | 0.565 | 0.374 |
|  | PC10 | 1.5 | 80.2 | 0.8±10.1 | -0.4±6.4 | 0.760 | 0.142 |
|  | PC11 | 1.4 | 81.6 | -0.0±7.0 | 1.7±1.3 | 0.401 | 0.338 |
|  | PC12 | 1.3 | 82.9 | -2.6±7.7 | 1.6±7.4 | 0.319 | 0.556 |
|  | PC13 | 1.2 | 84.2 | -1.0±7.2 | -1.3±2.3 | 0.889 | 0.056 |
|  | PC14 | 1.2 | 85.3 | 0.5±6.3 | 5.3±3.2 | **0.050** | 0.961 |
|  | PC15 | 1.1 | 86.4 | 3.9±5.4 | -0.9±7.3 | 0.237 | 0.748 |

The “*” symbol indicates significant differences between BBS-high and BBS-low groups (**p* < 0.2). *PC* principal component, *BBS* Berg balance scale.

**Supplementary Table S1-d.** Results of main principal component analysis in **sit to stand**.

| **Categories** | | **Explained Variance**  **(%)** | **Cumulative (%)** | **BBS High**  **(mean±SD)** | **BBS Low (mean±SD)** | ***p*** | **Cohan's *d*** |
| --- | --- | --- | --- | --- | --- | --- | --- |
| Paretic | PC1 | 56.7 | 56.7 | -45.8±4.9 | -57.5±6.3 | **0.171** | 2.073 |
|  | PC2 | 7.2 | 63.9 | 1.3±15.8 | -2.6±15.1 | 0.810 | 0.252 |
|  | PC3 | 5.2 | 69.0 | -6.4±14.0 | 1.0±22.3 | 0.229 | 0.397 |
|  | PC4 | 4.5 | 73.5 | 0.9±15.8 | -7.4±7.7 | 0.450 | 0.668 |
|  | PC5 | 3.7 | 77.3 | -1.6±13.7 | 3.5±12.7 | 0.372 | 0.386 |
|  | PC6 | 3.2 | 80.5 | -3.4±11.9 | 8.5±14.3 | 0.200 | 0.905 |
|  | PC7 | 2.8 | 83.3 | 3.4±10.3 | -6.4±14.7 | **0.051** | 0.772 |
|  | PC8 | 2.1 | 85.4 | 0.6±10.4 | -2.2±10.6 | 0.612 | 0.267 |
|  | PC9 | 1.9 | 87.3 | -0.9±11.4 | 0.4±6.2 | 0.796 | 0.142 |
|  | PC10 | 1.9 | 89.1 | 0.9±9.7 | -2.0±5.9 | 0.374 | 0.361 |
|  | PC11 | 1.8 | 91.0 | -0.3±9.4 | 1.7±9.0 | 0.667 | 0.217 |
|  | PC12 | 1.7 | 92.6 | 1.4±9.2 | -4.0±8.3 | 0.227 | 0.616 |
|  | PC13 | 1.3 | 93.9 | 1.2±9.2 | -2.4±7.6 | 0.240 | 0.427 |
|  | PC14 | 1.2 | 95.1 | 0.3±8.2 | -1.7±8.2 | 0.603 | 0.245 |
|  | PC15 | 1.1 | 96.2 | 1.0±8.2 | -2.4±4.2 | 0.318 | 0.522 |
|  | PC16 | 1.1 | 97.3 | 0.5±7.1 | -0.9±9.9 | 0.585 | 0.163 |
|  | PC17 | 1.0 | 98.3 | 1.0±8.0 | -2.4±7.3 | **0.163** | 0.444 |
| Non-paretic | PC1 | 54.4 | 54.4 | -47.9±6.3 | -50.8±15.6 | 0.384 | 0.244 |
|  | PC2 | 7.0 | 61.4 | -5.6±33.5 | 16.0±10.7 | **0.029** | 0.869 |
|  | PC3 | 5.9 | 67.2 | -2.7±25.7 | 7.3±6.9 | 0.388 | 0.531 |
|  | PC4 | 4.3 | 71.6 | 1.4±21.6 | -4.8±11.6 | 0.274 | 0.358 |
|  | PC5 | 3.8 | 75.4 | 0.7±8.8 | -4.2±14.5 | 0.493 | 0.409 |
|  | PC6 | 3.2 | 78.6 | -0.5±17.1 | 1.4±8.8 | 0.798 | 0.140 |
|  | PC7 | 2.7 | 81.3 | 0.8±7.3 | -1.1±11.5 | 0.803 | 0.197 |
|  | PC8 | 2.4 | 83.7 | -1.8±10.0 | 4.2±10.5 | 0.305 | 0.585 |
|  | PC9 | 2.2 | 85.9 | 0.3±9.7 | -0.1±9.7 | 0.923 | 0.041 |
|  | PC10 | 1.9 | 87.8 | 2.1±10.6 | -5.4±3.5 | **0.065** | 0.950 |
|  | PC11 | 1.8 | 89.5 | -0.1±9.8 | -0.9±8.2 | 0.865 | 0.089 |
|  | PC12 | 1.7 | 91.2 | -0.1±9.0 | -0.9±7.6 | 0.875 | 0.096 |
|  | PC13 | 1.6 | 92.8 | 0.0±8.5 | 0.0±4.2 | 0.997 | 0 |
|  | PC14 | 1.5 | 94.3 | -1.4±7.8 | 3.8±7.0 | 0.263 | 0.702 |
|  | PC15 | 1.3 | 95.6 | -2.1±7.8 | 4.5±5.6 | **0.038** | 0.972 |
|  | PC16 | 1.3 | 96.8 | 0.3±7.9 | -1.4±3.5 | 0.739 | 0.278 |
|  | PC17 | 1.2 | 98.1 | 0.4±7.7 | -0.6±2.2 | 0.810 | 0.177 |
|  | PC18 | 1.0 | 99.1 | -0.8±7.4 | 1.9±3.0 | 0.327 | 0.478 |

The “*” symbol indicates significant differences between BBS-high and BBS-low groups (**p* < 0.2). *PC* principal component, *BBS* Berg balance scale.

**Supplementary** **Table S2.** Definition of joint-specific motion for each axis of the kinematic data used in principal component analysis.

| **Kinematic categories** | | | **Joint movements** | | | |
| --- | --- | --- | --- | --- | --- | --- |
| Plane | Axis | Direction | Pelvis | Hip | Knee | Ankle |
| Sagittal plane | X | + | Posterior tilt | Flexion | Flexion | Dorsiflexion |
|  |  | - | Anterior tilt | Extension | Extension | Plantarflexion |
| Frontal plane | Y | + | Upward rotation | Adduction | Varus | Inversion |
|  |  | - | Downward rotation | Abduction | Valgus | Eversion |
| Horizontal plane | Z | + | Posterior rotation | Internal rotation | Internal rotation | Toe in |
|  |  | - | Anterior rotation | External rotation | External rotation | Toe out |
